# Dysregulated Hypothalamic–Pituitary–Adrenal Axis Is Associated With Increased Inflammation and Worse Outcomes After Ischemic Stroke in Diabetic Mice

**DOI:** 10.3389/fimmu.2022.864858

**Published:** 2022-06-16

**Authors:** Sehee Kim, Eun S. Park, Peng R. Chen, Eunhee Kim

**Affiliations:** Vivian L. Smith Department of Neurosurgery, McGovern Medical School, The University of Texas Health Science Center at Houston, Houston, TX, United States

**Keywords:** stroke, diabetes, HPA axis, inflammation, metyrapone

## Abstract

Diabetic patients have larger infarcts, worse neurological deficits, and higher mortality rate after an ischemic stroke. Evidence shows that in diabetes, the hypothalamic–pituitary–adrenal (HPA) axis was dysregulated and levels of cortisol increased. Based on the role of the HPA axis in immunity, we hypothesized that diabetes-dysregulated stress response exacerbates stroke outcomes *via* regulation of inflammation. To test this hypothesis, we assessed the regulation of the HPA axis in diabetic mice before and after stroke and determined its relevance in the regulation of post-stroke injury and inflammation. Diabetes was induced in C57BL/6 mice by feeding a high-fat diet and intraperitoneal injection of streptozotocin (STZ), and then the mice were subjected to 30 min of middle cerebral artery occlusion (MCAO). Infarct volume and neurological scores were measured in the ischemic mice. The inflammatory cytokine and chemokine levels were also determined in the ischemic brain. To assess the effect of diabetes on the stroke-modulated HPA axis, we measured the expression of components in the HPA axis including corticotropin-releasing hormone (CRH) in the hypothalamus, proopiomelanocortin (POMC) in the pituitary, and plasma adrenocorticotropic hormone (ACTH) and corticosterone. Diabetic mice had larger infarcts and worse neurological scores after stroke. The exacerbated stroke outcomes in diabetic mice were accompanied by the upregulated expression of inflammatory factors (including IL-1β, TNF-α, IL-6, CCR2, and MCP-1) in the ischemic brain. We also confirmed increased levels of hypothalamic CRH, pituitary POMC, and plasma corticosterone in diabetic mice before and after stroke, suggesting the hyper-activated HPA axis in diabetic conditions. Finally, we confirmed that post-stroke treatment of metyrapone (an inhibitor of glucocorticoid synthesis) reduced IL-6 expression and the infarct size in the ischemic brain of diabetic mice. These results elucidate the mechanisms in which the HPA axis in diabetes exacerbates ischemic stroke. Maintaining an optimal level of the stress response by regulating the HPA axis may be an effective approach to improving stroke outcomes in patients with diabetes.

## Introduction

Diabetes is an independent and modifiable risk factor for stroke (1). Approximately 30% of stroke patients have diabetes, and this number is even higher when patients with pre-diabetes are considered (2–4). Diabetes is also linked to poor stroke outcomes including higher mortality, larger infarction, and worse long-term recovery (5, 6). These clinical observations are supported by several experimental studies that have shown exacerbated ischemic brain injury and poor neurological outcomes in diabetic conditions following stroke (7–11). However, our understanding of the pathology and molecular mechanisms of diabetes-exacerbated stroke outcomes is limited.

The hypothalamus–pituitary–adrenal (HPA) axis is a complex neuroendocrine system that maintains physiologic homeostasis under stressful conditions (12). In response to stress stimuli, corticotropin-releasing hormone (CRH) is released from the paraventricular nucleus (PVN) of the hypothalamus. The CRH binds to CRHR, its receptor on the pituitary releasing adrenocorticotropic hormone (ACTH). Circulating ACTH stimulates glucocorticoid (GC) synthesis and secretion from the adrenal gland (13). The activation of the HPA axis is associated with poor stroke outcomes. Elevated plasma glucocorticoid levels correlate with higher rates of morbidity and mortality in humans (14–16) and increased infarct volume and poor functional recovery in the rodent models (17–21).

Dysregulated HPA axis is also linked to diabetes. Increased circulating GCs levels were observed in diabetic patients (22–24). Many studies have revealed that chronic stress with high GCs is associated with the onset of hyperglycemia or insulin resistance (25, 26). GCs further exacerbated hyperglycemia in patients with diabetes (27). In addition, continuous exposure to GCs is also associated with post-stroke hyperglycemia (28, 29). While this evidence suggests that a dysregulated HPA axis would be related to the unfavorable stroke outcomes, there is a lack of understanding of the regulation and impact of the HPA axis in stroke with diabetes.

GCs are widely used to treat inflammatory diseases by their anti-inflammatory activity (30, 31). However, accumulating evidence shows the controversial role of GCs in inflammation. GCs can increase certain parameters of the inflammatory response, primarily in the central nervous system (CNS) depending on the dose and chronicity of exposure (8, 32). For example, high GC levels did suppress mRNA levels of CX3CL1 and CX3CR1 (factors that restrain inflammatory responses), but increased the interleukin (IL)-1β mRNA expression in the hippocampus (33). Chronically enhanced plasma GCs increased the lipopolysaccharide-induced NFκB activation as well as the expression of pro-inflammatory factors including IL-1β and tumor necrosis factor (TNF)-α, while simultaneously decreasing the expression of anti-inflammatory factors in the frontal cortex and hippocampus of rats (33). As dysregulated inflammation is a critical pathology in ischemic stroke, the evidence suggests that GCs may play an important role in regulating inflammation in diabetic stroke.

In this study, we investigated the dysregulation of the HPA axis in diabetic conditions after stroke and tested whether inhibition of GC synthesis improves outcomes. In diabetic mice, we found hypothalamic CRH, pituitary POMC, and plasma corticosterone levels to be higher. These levels were associated with increased infarct volume, poorer neurological score, and higher inflammatory response. Finally, we found that metyrapone (an inhibitor of GC synthesis) demonstrated a protective effect after ischemic stroke with reduced IL-6 expression. These results suggest that regulation of the optimal level of the HPA axis would be important to minimize ischemic injury after stroke with diabetes.

## Materials and Methods

### Animals

All experiments performed with animals were approved by the Center for Laboratory Animal Medicine and Care (CLAMC) of the University of Texas Health Science Center at Houston. Male C57BL/6 mice were purchased from the Jackson Laboratory (Bar Harbor, ME) and bred at CLAMC. The facility monitors and maintains the temperature, humidity, and a 12-h light/dark cycle. A maximum of 5 mice were housed in a single cage with a ventilating system with freely accessible food and water in each cage. Mice were randomized and blinded to specific diets, treatments, and surgery. In total, 101 mice (29 mice for ND/veh and 72 mice for DD/STZ) were used in this study. Fourteen mice were in the “prior to stroke” group without MCAO (5 mice for ND/veh and 9 mice for DD/STZ), and 87 mice (24 for ND/veh and 63 for DD/STZ) were subjected to MCAO surgery. After excluding the mice that did not meet MCAO criteria or died before the end point, 74 mice were assigned to the different groups (14 for prior to stroke, 5 for ND/veh and 9 for DD/STZ; 20 for 1 day post-stroke, 10 for ND/veh and 10 for DD/veh; 17 for 3 days post-stroke, 7 for ND/veh and 10 for DD/STZ; 23 for metyrapone effect, 10 for DD/STZ/veh and 13 for DD/STZ/metyrapone).

### Induction of Diabetes in Mice

Six-week-old male C57BL/6 mice were fed a diabetic diet (DD; 60% kcal from fat, S3282, Bio-Serv, Frenchtown, NJ). Three weeks after the diet, vehicle or a low dose of streptozotocin (STZ) (40 mg/kg/day; Cat. No. S0130, Millipore Sigma) was administered by intraperitoneal (i.p.) injection for 5 consecutive days. The DD was continuously fed during and after the STZ injection for 8 weeks. Mice fed normal chow (ND) with vehicle (citrate buffer) injection were used as the normal control group.

### Blood Glucose Measurement and Glucose Tolerance Test

To confirm the induction of diabetic condition in mice, blood glucose measurement and glucose tolerance test (GTT) were performed in overnight fasted mice after 7 weeks of the diet. Glucose levels were measured in the blood from a tail snip using a glucometer (Contour, Bayer, Germany). For GTT, the overnight fasted mice were i.p. injected with 2 g/kg of D-glucose, and blood glucose levels were measured at 15, 45, and 120 min after the glucose injection.

### Transient Middle Cerebral Artery Occlusion

The normal and diabetic mice were subjected to MCAO by the intraluminal thread method (10, 34, 35). Isoflurane anesthetized mice were placed prone. To monitor cerebral blood flow (CBF), a fiber-optic probe was attached to the parietal bone (2 mm posterior and 5 mm lateral to the bregma) using the vet bond and connected to a Laser-Doppler Flowmeter (Periflux System 5010; Perimed, Järfälla, Sweden). The mice were turned from prone to supine, and then approximately 1.5 cm of incision was made on the mouse neck to expose the right common carotid artery. A 6-0 silicone-coated black monofilament surgical suture (Cat. No. 602156PK10, Doccol Co., Redland, CA, USA) was inserted into the exposed external carotid artery and advanced into the internal carotid artery, and wedged into the Circle of Willis to obstruct the origin of the MCA. The filament was left in place for 30 min and then removed for blood reperfusion. During the entire MCAO procedure, the mouse body temperature was maintained at 37 ± 0.5°C using a Masterflex pump and thermistor temperature controller (Cole-Parmer, Vernon Hills, IL, USA). Only mice exhibiting CBF with more than 80% of reduction compared to pre-ischemic baseline during MCAO and recovering greater than 80% of the baseline by 10 min of reperfusion were included in the study. Seven out of the 24 ND/veh and 18 out of the 63 DD/STZ mice did not meet the criteria. Therefore, the MCAO success rate was similar between the ND/veh (70.8%) and DD/STZ (71.4%) groups. In the mice with successful MCAO (17 for ND/veh and 45 for DD/STZ), all ND/veh mice survived, but 2 mice in the DD/STZ group died before the endpoint (3 days post-stroke). Therefore, the mortality rate is 0% for ND/veh and 4.4% for DD/STZ.

### Plasma ACTH and Corticosterone Measurement

For ACTH and corticosterone measurement, mouse trunk blood was collected into tubes containing heparin. The collected blood samples were immediately centrifuged at 3,000 rpm for 10 min, and the plasma samples were stored at −80°C until the analysis. Plasma ACTH levels were detected by an ACTH ELISA kit (Cat. No. ab263880, Abcam, Cambridge, UK), and the plasma corticosterone levels were determined using a corticosterone ELISA kit (Cat. No. K014, Ann Arbor, Michigan, USA) according to the manufacturer’s procedure.

### Metyrapone Treatment

Diabetic mice were administered either vehicle (PBS) or 100 mg/kg metyrapone (M2696, Millipore Sigma) by i.p. injection after ischemic stroke (right after reperfusion, and 1 day and 2 days post-MCAO). Mice were sacrificed at 3 days post-MCAO.

### Tissue Collection and Brain Section Strategy

Brains were collected, frozen, and sectioned in a cryostat (Leica Biosystem Inc.) as previously described (10). Briefly, sections were collected between roughly +3.1 mm and −4.1 mm from bregma. For injury measurement, 20 μm thickness of sections were collected at 600-μm intervals on slide glass. Brain tissues between the 600-μm intervals were sectioned and cut in half for each hemisphere and collected to determine mRNA and protein levels. To collect the hypothalamus, the hypothalamic area was dissected from the coronal-sectioned brain tissues. We cut the midline and separately collected the contralateral and ipsilateral sides of hypothalamus. The pituitary gland is located in the shallow depression, midline of the dorsal surface of the basisphenoid skull bone. Mice were sacrificed to collect the pituitary gland, and after the skull was removed, the whole brain was collected with fine curved forceps.

### Infarct Volume and Swelling Measurement

Infarct volume and hemispheric swelling were measured using the Zen software (Zeiss, Germany) by a method described previously (36, 37). Infarct volume was determined as an integrating volume of each section obtained by infarct area (mm^2^) multiplied by the distance between adjacent sections (0.6 mm). The final infarct volume was calculated after subtracting the volume difference between the contralateral and ipsilateral sides to correct for swelling. Percent hemispheric swelling (% HS) was calculated using the difference in volume between the two hemispheres and then dividing it by the contralateral hemisphere volume according to the following formula: % HS = [(ipsilateral volume − contralateral volume)/contralateral volume] × 100 (11). Representative brain sections were stained using cresyl violet. Briefly, sections were fixed with 10% buffered formalin phosphate (SF100-4, Fisher Scientific, Hampton, NH) on slides and stained with Cresyl Violet Solution (0.1%, ab246816, Abcam, Waltham, MA) for 8 min. The sections were rinsed quickly in distilled water and swiftly dehydrated in absolute alcohol. Stained sections dehydrated in xylene and mounted with Permount mounting medium (SP15-100). Images were captured under a microscope (Zeiss SteREO Discovery, V12).

### Neurological Deficit Measurement

Neurological scores were evaluated at 2 and 24 h of reperfusion based on the Zea Longa method (38). The scores were graded from 0 to 5: 0 = no observed deficit, 1 = forelimb flexion when lifted by the tail, 2 = consistently reduced resistance to lateral push, 3 = unilateral circling toward the paretic side, 4 = ambulation inability or difficulty, and 5 = dead.

### Gene Expression Analyses by Real-Time RT-PCR

To isolate total RNA, tissues were homogenized in Tri reagent (MRC, OH, USA). The lysates were then precipitated with isopropanol, and the pellets were washed with 70% ethanol, air-dried, and dissolved in sterile diethylpyrocarbonate (DEPC) water. The concentration and purity of total RNAs were determined with a NanoDrop spectrophotometer (NanoDrop Technologies, Inc., Wilmington, DE, USA). One microgram of total RNA was reverse transcribed using oligo (dT) primers and Superscript II reverse transcriptase (Life Technologies, Inc. Carlsbad, CA) according to the manufacturer’s protocol. The PCR reaction was performed by 7500 Fast Real-Time PCR System (Applied Biosystem, Foster City, CA) using amfiSuer qGreen Q-PCR Master Mix (GenDepot, Katy, TX) and specific PCR primers for CRH (For: CCTGGGGAAT CTCAACAGAA, Rev: AACACGCGGAAAAAGTTAGC), POMC (For: TGTACCCCAACGTTG CTGAG, Rev: AGGACCTGCTCCAAGCCTAA), IL-6 (For: TGGTACTCCAG AAGACCAGAGG, Rev: AACGATGATGCACTTGCAGA, IL-1β (For: GCACACCCACCCTGCA, Rev: ACCGCTTT TCCATCTTCTTCTT), TNF-α (For: ATGGCCTCCCTCTCATCAGT, Rev: TTTGCTACGACGT GGGCTAC), CCR2 (For: ACAGCTCAGGATTAACAGGGACTTG, Rev: ACCACTTGCATGCA CACATGAC), MCP-1 (For: GCATCCACGTGTTGGCTCA, Rev: CTCCAGCCTACTCATTGG GATCA), and GAPDH (For: TTGATGGCAACAATCTCCAG, Rev: CGTCCCGTAGACAAAATGGT).

### Statistical Analyses

The sample size for our study was calculated based on the previous study using ND/veh and DD/STZ mice (11). We needed a minimum of 7 mice per group to reach a power of 0.80 at a significance level of <0.05 predicting a 40% difference in mean and a 25% standard deviation at the 95% confidence level. GraphPad Prism (GraphPad Software, San Diego, California, USA) was used for data analysis. Results are presented as mean ± standard deviation (SD). Student’s *t*-test was used to compare means between the two groups. Multiple comparisons were performed using analysis of variance (ANOVA) followed by a post-hoc Bonferroni’s multiple comparison test. Differences were considered statistically significant at *p* < 0.05.

## Results

### Diabetic Mice Display Exacerbated Brain Injury and Neurological Deficit After Ischemic Stroke

**Figure 1:**
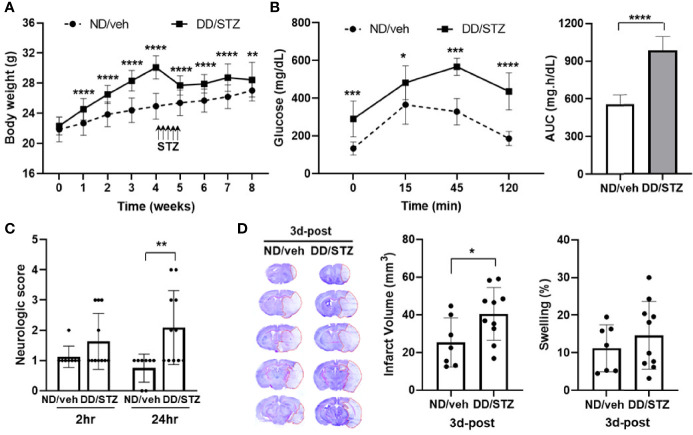
Increased stroke injury and neurological deficits in diabetic mice. **(A)** Body weight gain in diabetic mice (DD/STZ) and normal mice (ND/veh) for 8 weeks. **(B)** Glucose tolerance test (GTT) and area under the curve (AUC). Overnight-fasted mice were given an intraperitoneal (i.p.) injection of glucose. Blood samples were taken from the tail vein at the times indicated. *n* = 10/group (10 for ND/veh, 10 for DD/STZ). **(C)** Neurological scores for mice at 2 and 24 h after stroke. *n* = 8–11/group (8 for ND/veh, 11 for DD/STZ). **(D)** Mice were subjected to 30 min of MCAO, and infarct volume and swelling were measured in coronal sections of mouse brains at 3 days post-stroke (3d-post). *n* = 7–10/group (7 for ND/veh, 10 for DD/STZ). Data are presented as means ± SD; *p < 0.05, **p < 0.01, ***p < 0.001, ****p < 0.0001 vs. ND/Veh. ND/veh, mice fed normal chow and injected vehicle; DD/STZ, mice fed DD and injected STZ.

We induced a diabetic condition in C57BL/6 mice by a combination of feeding DD and injection of STZ. The body weights were significantly increased in mice by feeding DD compared to normal chow (ND). The body weight was decreased in the DD/STZ group by STZ injection and sustained without an increase for the next 4 weeks after STZ injection even though the mice were continuously fed DD. At the endpoint, DD/STZ mice showed a slight but significant increase in body weight compared to ND/veh (**Figure 1A**). To confirm the diabetic condition in DD/STZ mice, we performed the glucose tolerance test (GTT). Diabetic mice showed significantly higher blood glucose levels compared to normal (ND/veh) mice. GTT confirmed that glucose clearance was also significantly delayed in DD/STZ mice (**Figure 1B**). One week after the GTT, mice were subjected to MCAO, and the neurological scores were measured. Compared to ND/veh mice, DD/STZ mice showed higher neurological scores at 1 day after stroke (**Figure 1C**) and significantly larger infarct size at 3 days post-stroke (**Figure 1D**). The swelling was slightly higher in DD/STZ mice, but this difference was not significant (**Figure 1D**). These results indicate that the diabetic condition exacerbates brain injury and neurological deficit in mice after ischemic stroke.

### Diabetic Condition Increase Inflammation After Ischemic Stroke

**Figure 2:**
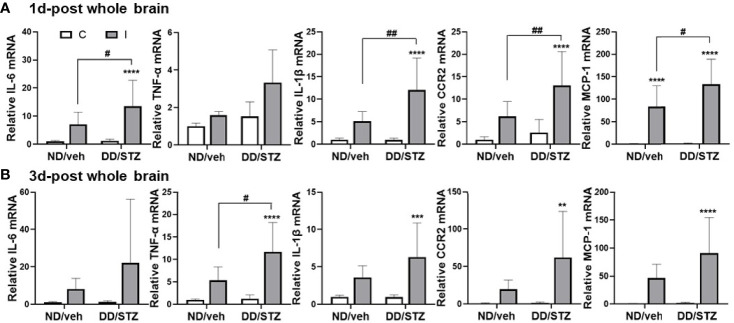
Increased inflammatory cytokines and chemokines in the ischemic brain of diabetic mice. **(A, B)** Real-time PCR analyses of IL-1β, IL-6, TNF-α, CCR2, and MCP-1 mRNA expression in the ischemic brain at 1 day (1d-post) **(A)** or 3 days post-stroke (3d-post) **(B)**. Each gene expression level was normalized by GAPDH. The mean value of contralateral side of ND/veh at pre was regarded as 1, and we calculated the relative values for the others. *n* = 7–10/group (1d-post, 10 for ND/veh, 10 for DD/STZ; 3d-post, 7 for ND/veh, 10 for DD/STZ). Data are presented as means ± SD; **p < 0.01, ***p < 0.001, ****p < 0.0001 vs. ND/Veh, ^#^p < 0.05, ^##^p < 0.01, vs. 1 days post-stroke. Two-way ANOVA and Bonferroni test; ND/veh, mice fed normal chow and injected vehicle; DD/STZ, mice fed HFD and injected STZ; C, contralateral; I, ipsilateral.

To determine the effect of diabetes on stroke-induced inflammation, we assessed the mRNA level of inflammatory mediators in the ischemic brain. We confirmed that brain IL-6, IL-1β, TNF-α, CCR2, and MCP-1 expression levels were not significantly different between ND/veh and DD/STZ mice prior to stroke (data not shown). Levels of these inflammatory factors, except TNF-α, significantly increased in the ischemic brain in DD/STZ mice at 1 day post-stroke (**Figure 2A**). At 3 days post-ischemic brain, these pro-inflammatory factors were still overall higher in DD/STZ mice (**Figure 2B**). TNF-α expression was not significantly elevated in the ischemic brain of DD/STZ mice at 1 day post-stroke; however, we observed the delayed increase in TNF-α in DD/STZ mice at 3days post-stroke (**Figures 2A, B**).

### Diabetic Condition Dysregulates HPA Axis Prior to and After Stroke

**Figure 3:**
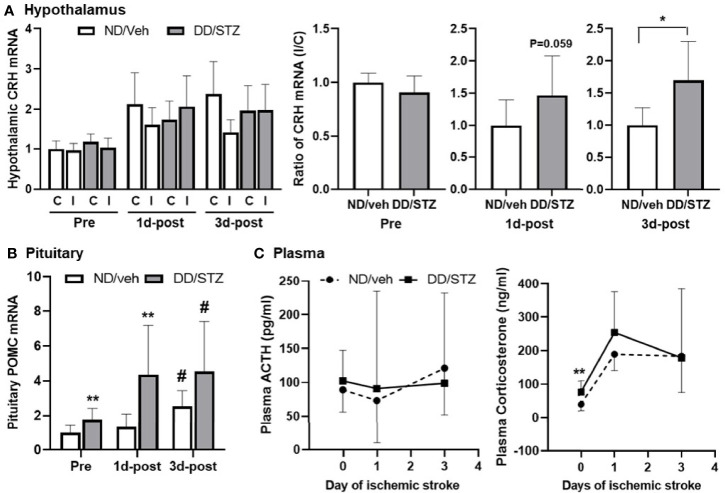
Enhanced expression of HPA components and plasma glucocorticoids in diabetic mice before and after ischemic stroke. **(A)** Real-time PCR analysis of CRH mRNA levels in the hypothalamus. Each gene expression level was normalized by GAPDH. The mean value of contralateral of ND/veh (hypothalamus) at pre or ND/veh (for the ratio) was regarded as 1, and we calculated the relative values for the others. Data are presented as means ± SD. *p < 0.05 vs. ND/Veh, Student’s *t*-test. **(B)** POMC mRNA in the pituitary of ND/Veh and DD/STZ mice. Each gene expression level was normalized by GAPDH. The mean value of ND/veh at pre was regarded as 1, and we calculated the relative values for the others. Data are presented as means ± SD. *p < 0.05, **p < 0.01 vs. ND/Veh. ^#^ *p* < 0.05 vs. Pre. Two-way ANOVA and Bonferroni test. **(C)** Plasma ACTH and corticosterone levels in the mice before (pre) and after stroke, 1 day (1d-post) or 3 days post-stroke (3d-post). *n* = 5–10/group (prior to stroke, 5 for ND/veh, 9 for DD/STZ; 1d-post, 10 for ND/veh, 10 for DD/STZ; 3d-post, 7 for ND/veh, 10 for DD/STZ). Each value is the mean ± SD. **p < 0.01 vs. ND/Veh, Student’s *t*-test; ND/veh, mice fed normal chow and injected vehicle; DD/STZ, mice fed HFD and injected STZ; C, contralateral; I, ipsilateral.

To investigate the effect of diabetic condition on the HPA axis, we compared the expression levels of components in the HPA axis between normal (ND/veh) and diabetic (DD/STZ) mice before and after stroke. Gene expression of hypothalamic CRH was similar in ND/veh and DD/STZ mice prior to stroke. The hypothalamic CRH levels in both ND/veh and DD/STZ mice tend to increase after stroke, although the overall statistical analysis did not show any significance. However, we noted that ND/veh mice showed lower levels of CRH in the ischemic side of the hypothalamus compared to the non-ischemic side, while the DD/STZ mice did not exhibit lower CRH in the ischemic side of the hypothalamus. The ratio between the ischemic and non-ischemic sides was significantly higher in DD/STZ mice (**Figure 3A**). In the pituitary gland, we found significantly elevated mRNA levels of POMC (the precursor of ACTH) in the anterior pituitary in DD/STZ mice compared to ND/veh before stroke. The POMC gene expression was further increased in DD/STZ mice at 1 day post-stroke while the levels in ND/veh mice remained similar to the levels in pre-ischemia and sustained until 3 days post-stroke (**Figure 3B**). The pituitary POMC gene levels were also increased in ND/veh mice at 3 days post-stroke, but the levels were still lower than those in DD/STZ mice (**Figure 3B**). Despite the upregulated POMC in the pituitary, plasma ACTH levels were not significantly higher in DD/STZ mice before and after stroke. While the ACTH levels varied a lot in mice after stroke, the average levels were still similar between the groups (**Figure 3C**). However, plasma corticosterone (the main GCs in rodents) levels were significantly higher in the DD/STZ group compared to the ND/veh group before stroke. Stroke increased corticosterone levels in both ND/veh and DD/STZ mice. The levels were still higher in DD/STZ at 1 day post-stroke although, there was no statistical significance. The increased plasma corticosterone levels were slightly decreased in DD/STZ at 3 days post-stroke compared to 1 day post-stroke while similar levels of corticosterone were maintained in the ND/veh group at both time points (**Figure 3C**). Overall, these results suggest enhanced stress response in diabetic conditions.

### Metyrapone Reduces Diabetes-Exacerbated Ischemic Injury

**Figure 4:**
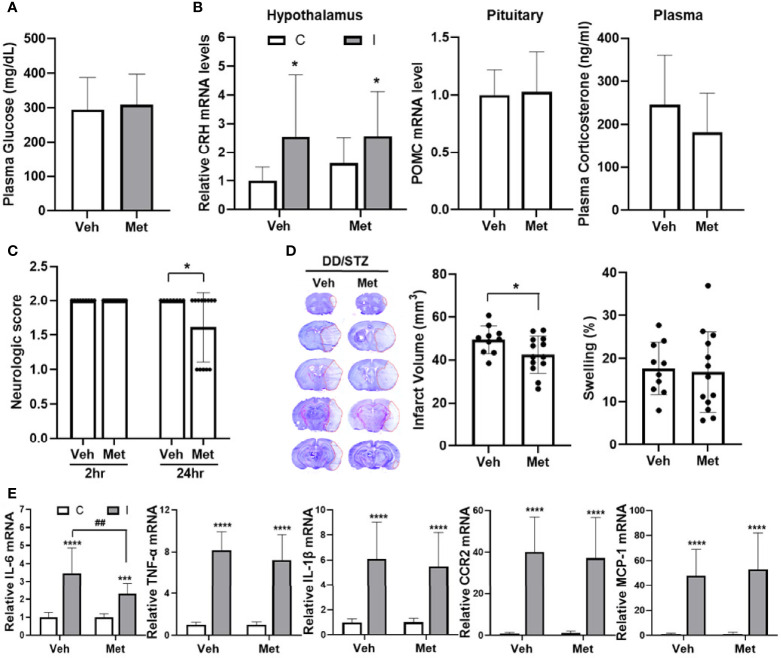
Reduce ischemic injury in diabetic mice by inhibition of GC synthesis. Mice were treated with either vehicle (veh) or metyrapone (100 mg/kg) by intraperitoneal (i.p.) injection daily for 3 days after ischemic stroke. **(A)** Plasma glucose levels. Glucose levels were determined by a glucometer in the plasma isolated from trunk blood at 3 days post-stroke. **(B)** Gene expression of CRH and POMC in the hypothalamus and pituitary, and plasma corticosterone levels. Each gene expression level was normalized by GAPDH. The mean value of contralateral of veh (hypothalamus) or veh (pituitary) was regarded as 1, and we calculated the relative values for the others. Plasma corticosterone levels were measured in the diabetic mice at 3 days post-stroke. Data are presented as mean ± SD. **(C)** Neurological scores in DD/STZ mice treated with veh or metyrapone. **(D)** Infarct volume and swelling were measured in coronal section mouse brains. Data are presented as mean ± SD. *p < 0.05 vs. Veh, Student’s *t*-test. **(E)** Gene expression of inflammatory mediators in the mouse brain at 3 days post-stroke. Each gene expression level was normalized by GAPDH. The mean value of contralateral of veh was regarded as 1, and we calculated the relative values for the others. *n* = 10–13/group (10 for vehicle, 13 for metyrapone). Data are presented as mean ± SD, ***p < 0.001, ****p < 0.0001 vs. C; ^##^ *p* < 0.01 vs. I of Veh, two-way ANOVA; C, contralateral; I, ipsilateral. Veh, DD/STZ mice treated with veh; Met, DD/STZ mice treated with metyrapone.

To address the effect of GC inhibition on diabetic-exacerbated ischemic injury, we used metyrapone, an inhibitor of GC synthesis (39). DD/STZ mice were treated with metyrapone by i.p. injection (100 mg/kg) starting immediately after MCAO, 1 day, and 2 days post-stroke. At 3 days post-stroke, plasma glucose levels were similar between DD/STZ treated with vehicle and metyrapone (**Figure 4A**). The post-stroke treatment of metyrapone reduced plasma corticosterone levels in DD/STZ mice without significance (**Figure 4B**). However, metyrapone did not affect the hypothalamic CRH and pituitary POMC expression (**Figure 4B**). We confirmed that metyrapone ameliorated the neurological deficits induced in the DD/STZ mice after ischemic stroke (**Figure 4C**). The metyrapone-treated DD/STZ mice also showed a small but significant reduction of infarct volume, but not swelling (**Figure 4D**). We also confirmed the effects of metyrapone on the inflammatory response of the ischemic brain. Only IL-6 mRNA levels were significantly reduced in the ischemic brain of metyrapone-treated DD/STZ mice compared to vehicle-treated mice, while the levels of other markers (IL-1β, TNF-α, CCR2, and MCP-1) were not different between the groups (**Figure 4E**).

## Discussion

Although diabetes is a well-known risk factor for the incidence and poor outcomes after stroke, the mechanisms by which diabetes exacerbates stroke pathophysiology are a topic of ongoing investigation. To determine the role of HPA axis in ischemic stroke under diabetic condition, we performed MCAO in diabetic mice generated by the combination of feeding DD and injection of STZ. Previous studies have shown that the DD/STZ mice exhibited several features of type I and type II diabetes, including hyperglycemia and insulin resistance, in rodent models (11, 40, 41). In the DD/STZ mice, we confirmed the significantly higher glucose levels (**Figure 1B**). Insulin resistance is one of the hallmarks of type II diabetes, the most common type of diabetes. The Homeostasis Model Assessment of Insulin Resistance (HOMA-IR) is a useful method for evaluating insulin resistance. However, since the beta cells were artificially destroyed by STZ leading to basal levels of plasma insulin in DD/STZ mice (11), the HOMA-IR (which is determined by fasting glucose and insulin levels) would not be accurate in showing insulin resistance in mice. Therefore, we used GTT to test for insulin resistance and confirmed that there is delayed glucose clearance in DD/STZ mice compared to control mice (**Figure 1B**). The hyperglycemia and delayed glucose clearance confirmed that a diabetic condition was induced in the DD/STZ mice.

Previous studies using experimental animal models showed that dysregulated inflammatory responses were associated with poor stroke outcomes in diabetic conditions. Upregulation of inflammatory markers including IL-6, IL-1β, and monocyte chemoattractant protein (MCP)-1 was observed in the brains of diabetic mice or rats following transient MCAO (42, 43). Higher activation of NFκB was also shown to mediate the increased pro-inflammatory response in the ischemic brain in diabetic conditions (43). Consistently, the current study confirmed a worse neurological scores and a larger infarct size in diabetic mice after stroke (**Figures 1C, D**). These diabetes-induced worsening of stroke outcomes were accompanied by the increased inflammatory response in the ischemic brain. Stroke-induced cytokines (IL-6, TNF-α, and IL-1β) and MCP-1 and CCR2 mRNA levels were significantly higher in the diabetic brain compared to control at 1 day after MCAO, and this increased expression was sustained until 3 days post-stroke (**Figure 2**). Unfortunately, we did not test the neurological score at 3 days post-stroke. However, previous studies have shown that neurological deficits increased rapidly in the acute phase of stroke (1 day post-stroke) and showed similar trends between 1 day and 3 days post-stroke. Neurological scores correlate with injury size (44–47), and we assume that the neurological scores at 3 days post-stroke would have a similar trend to those at 1 day post-stroke. In conjunction with findings from other studies, our data demonstrate that diabetes worsened stroke outcomes, and the pro-inflammatory response might contribute to the diabetes-induced exacerbation.

Dysregulated HPA axis and increased stress response have been reported in stroke patients (14, 48, 49). Experimental studies also showed that plasma corticosterone is significantly higher in ischemic injury in both global and transient focal ischemia in rats (20, 29). In parallel, clinical and preclinical lines of evidence demonstrated that diabetes alters HPA axis and induces hypersecretion of glucocorticoids (50–52). However, it has not been shown how the HPA axis is regulated in diabetic conditions after stroke and whether the altered HPA axis affects stroke outcome. In the current study, we found the overall increased hypothalamic CRH, pituitary POMC, and plasma corticosterone levels in both normal and diabetic mice after stroke. Notably, the stroke-induced expression of HPA components was further increased in diabetic mice compared to normal mice (**Figure 3**), suggesting that diabetes induces hyper-activation of the HPA axis in response to ischemic stroke.

Although we observed a significant increase in POMC in the pituitary of diabetic mice before and after stroke, the plasma ACTH levels were not significantly altered by stroke and diabetes (**Figure 3C**). Previous studies have shown that ACTH levels were relatively unchanged throughout reperfusion after MCAO, and the changes were also observed at earlier time points after stroke (2 h or 4 h post-stroke) (53, 54). Therefore, the time points in our study (1 day or 3 days post-MCAO) may not be ideal for comparing the ACTH levels in the mouse groups. In addition, HPA axis activity has a circadian rhythm that is driven by the regulation of complex clock genes (55–57). While the plasma concentration of ACTH and GCs fluctuates a lot, most rodent species show that the circadian rhythms peak during the night (58, 59). Therefore, collecting samples at the same time point would be ideal. However, there were practical difficulties in conducting MCAO surgery. For instance, it is limited to performing the MCAO surgery during the night. Also, the surgical procedure requires an absolute amount of time (at least 1 h per mouse). In this study, we collected all samples between 12 p.m. and 5 p.m.; however, there should be an impact of the hormonal fluctuation and circadian rhythm in our results. The overall moderate significance of HPA axis between the groups may be affected by that.

The detrimental impact of GCs in ischemic stroke has been shown in previous studies. GC treatment exacerbated neuronal cell death and brain damage in the mice after stroke (29). In diabetic patients, increased plasma cortisol levels were associated with poor stroke outcomes (60). Supportively, we found that the hyper-activated HPA axis in diabetic mice was accompanied by worse stroke outcomes and increased inflammatory response (**Figures 1** and **2**). To determine the role of the HPA axis in diabetes-exacerbated ischemic injury, we used metyrapone, an inhibitor of glucocorticoid synthesis. The post-stroke injection of metyrapone significantly improved neurological scores and decreased infarct volumes in the DD/STZ mice (**Figures 4C, D**). The beneficial effect of metyrapone on stroke outcomes in diabetic mice is consistent with the results seen in normal mice in the previous studies (20, 61, 62).

While our data show the beneficial effect of metyrapone in diabetes-exacerbated ischemic injury, the impact of metyrapone was not dramatic, as we only observed ~20% reduction of infarct (**Figure 4D**). The moderate efficacy of metyrapone in our study is likely due to the treatment approach. The short-term treatment of metyrapone (immediately after MCAO, and 1 day and 2 days post-MCAO) may not be able to significantly block GC synthesis. Also, there was a 1-day interval between the last metyrapone treatment (2d-post) and sample collection (3d-post), and the efficacy of metyrapone in reducing GC synthesis could have decreased during that time. In addition, metyrapone also inhibits aldosterone, which may have an additional effect on ischemic injury, as shown in previous studies (63–66). Further studies are required to test whether a higher dose and/or longer treatment duration of metyrapone can improve efficacy and whether the beneficial effect of metyrapone is through another mechanism rather than the reduction of GC synthesis. Additional studies using more specific methods to inhibit the activation of HPA axis are also suggested.

Classically, GCs have been considered as anti-inflammatory mediators (67); however, accumulating lines of evidence have shown that the effect of GCs on inflammation is dependent on the dose, exposure timing, and/or duration (68–70). In particular, GCs have been shown to be pro-inflammatory in CNS injury. GCs increased the number of inflammatory cells (granulocytes and microglia/macrophage) and IL-1β and TNF-α in the injured hippocampal area by kainic acid, an excitotoxic agent (32). Increased GCs and dysregulated HPA axis are linked to increased inflammation in the ischemic brain (71–74). Furthermore, myeloid GC signaling regulates the inflammatory response, subsequently contributing to neuronal injury following stroke (75). This study found that the diabetes-induced increase in HPA activity coincided with a pro-inflammatory response in the ischemic brain (**Figures 2** and **3**). Furthermore, we also confirmed that inhibition of GC synthesis by metyrapone reduced IL-6 expression in the ischemic brain of diabetic mice (**Figure 4**). Therefore, our study supports the pro-inflammatory potency of GCs.

The critical role of IL-6 in ischemic stroke has been shown. IL-6 is an early inflammatory signal in the ischemic brain (76) and correlates to the stroke severity (77, 78). Besides GC’s role in regulating IL-6 expression (79), the correlation between plasma IL-6 and cortisol levels has also been shown in acute stroke (48). Interestingly, IL-6 is involved in both pro- and anti-inflammatory responses (80). Reduction of IL-6 expression, but not the other cytokines, in the ischemic brain of diabetic mice treated with metyrapone (**Figure 4E**) indicates that IL-6 may be a critical cytokine in mediating the impact of an altered HPA axis in ischemic stroke. In addition to the link with IL-6, GCs have been involved in the regulation of leukocyte counts in stroke animal models and patients (81, 82). Another study also showed the increased infiltration and activation of neutrophils and lymphocytes in ischemic brain in type II diabetic mice (83). However, there is a lack of information regarding the link between the HPA axis and leukocyte trafficking in ischemic stroke with diabetes. Further studies should be conducted to detail how HPA axis regulates the inflammatory response and the trafficking of immune cells in diabetes-exacerbated ischemic stroke.

Taken together, our study demonstrates that DD/STZ-induced diabetes exacerbated ischemic injury and neurological scores in mice after stroke. The diabetes-exacerbated stroke outcomes were associated with increased inflammatory response and activation of the HPA axis. Finally, we confirmed that inhibition of GC synthesis reduced the diabetes-increased infarct size with reduced IL-6 expression. Our study demonstrates that diabetes-dysregulated stress response contributes to stroke injury *via* regulating the inflammatory response. Therefore, modulation of stress response might be an effective therapeutic strategy for stroke patients with diabetes.

## Data Availability Statement

The raw data supporting the conclusions of this article will be made available by the corresponding author, without undue reservation.

## Ethics Statement

The animal study was reviewed and approved by the Institutional Animal Care and Use Committee (IACUC) of the University of Texas Health Science Center at Houston, in accordance with the IACUC, National Institutes of Health, and ARRIVE (Animal Research: Reporting of *In Vivo* Experiments) guidelines.

## Author Contributions

SK: conducting experiments, data analysis, and writing the manuscript. ESP: conducting experiments, data analysis, and editing the manuscript. PRC: conception and editing the manuscript. EK: conception, supervision, design, data analysis, and writing the manuscript. All authors contributed to the article and approved the submitted version.

## Funding

This work was supported by the Weatherhead Foundation.

## Conflict of Interest

The authors declare that the research was conducted in the absence of any commercial or financial relationships that could be construed as a potential conflict of interest.

## Publisher’s Note

All claims expressed in this article are solely those of the authors and do not necessarily represent those of their affiliated organizations, or those of the publisher, the editors and the reviewers. Any product that may be evaluated in this article, or claim that may be made by its manufacturer, is not guaranteed or endorsed by the publisher.
